# Potential Modulation of Vascular Function by Nitric Oxide and Reactive Oxygen Species Released From Erythrocytes

**DOI:** 10.3389/fphys.2018.00690

**Published:** 2018-06-07

**Authors:** Joseph M. Rifkind, Joy G. Mohanty, Enika Nagababu, Maria T. Salgado, Zeling Cao

**Affiliations:** ^1^Department of Anesthesiology and Critical Care Medicine, Johns Hopkins University School of Medicine, Baltimore, MD, United States; ^2^National Institute on Aging, National Institutes of Health, Baltimore, MD, United States

**Keywords:** erythrocytes, vasculature, nitric oxide, reactive oxygen species, superoxide, hypoxia

## Abstract

The primary role for erythrocytes is oxygen transport that requires the reversible binding of oxygen to hemoglobin. There are, however, secondary reactions whereby the erythrocyte can generate reactive oxygen species (ROS) and nitric oxide (NO). ROS such as superoxide anion and hydrogen peroxide are generated by the autoxidation of hemoglobin. NO can be generated when nitrite reacts with hemoglobin forming an HbNO^+^ intermediate. Both of these reactions are dramatically enhanced under hypoxic conditions. Within the erythrocyte, interactions of NO with hemoglobin and enzymatic reactions that neutralize ROS are expected to prevent the release of any generated NO or ROS. We have, however, demonstrated that partially oxygenated hemoglobin has a distinct conformation that enhances hemoglobin-membrane interactions involving Band 3 protein. Autoxidation of the membrane bound partially oxygenated hemoglobin facilitates the release of ROS from the erythrocyte. NO release is made possible when HbNO^+^, the hemoglobin nitrite-reduced intermediate, which is not neutralized by hemoglobin, is bound to the membrane and releases NO. Some of the released ROS has been shown to be transferred to the vasculature suggesting that some of the released NO may also be transferred to the vasculature. NO is known to have a major effect on the vasculature regulating vascular dilatation. Erythrocyte generated NO may be important when NO production by the vasculature is impaired. Furthermore, the erythrocyte NO released, may play an important role in regulating vascular function under hypoxic conditions when endothelial eNOS is less active. ROS can react with NO and, can thereby modulate the vascular effects of NO. We have also demonstrated an inflammatory response due to erythrocyte ROS. This reflects the ability of ROS to react with various cellular components affecting cellular function.

## Introduction

The primary role of the erythrocyte is the transport of oxygen through the circulatory system and the delivery of oxygen to the tissues. Effective oxygen transport requires that the erythrocytes contain a major fraction of the required body oxygen and come into intimate contact with all the tissues of the body. The close contact of erythrocytes in the capillaries to the body tissues opens up the possibility that compounds produced by secondary reactions involving hemoglobin can also be transferred to the tissues and thereby influence the proper function of the organism.

**FIGURE 1:**
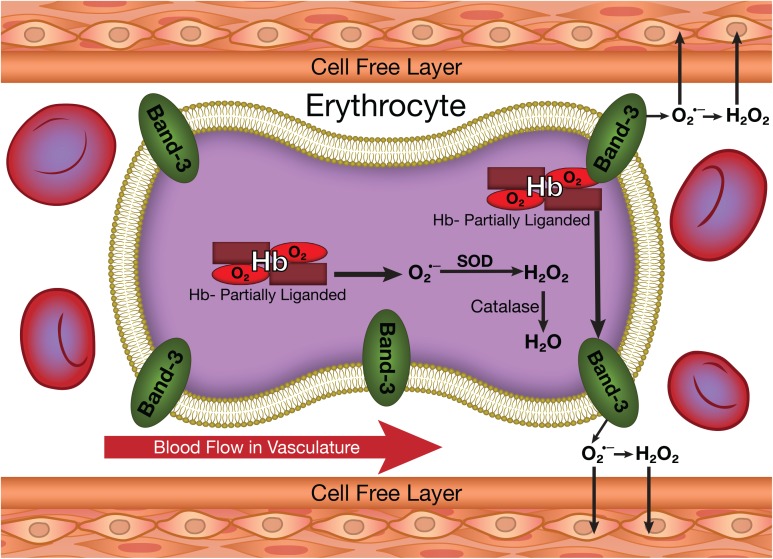
The production of superoxide and other ROS in erythrocytes and their potential transfer to the vasculature.

One of these secondary reactions of hemoglobin involves its autoxidation (**Figure 1**) resulting in the reduction of oxygen to superoxide (Levy et al., 1991). Hemoglobin is relatively stable but does slowly undergo autoxidation. This reaction, which is very slow for fully oxygenated hemoglobin, is increased by several orders of magnitude for partially oxygenated hemoglobin (Abugo and Rifkind, 1994; Balagopalakrishna et al., 1996). Reactions of superoxide generate other reactive oxygen species (ROS) including hydrogen peroxide (**Figure 1**) and hydroxyl radicals (Nagababu et al., 2003a; Nagababu and Rifkind, 2004). These ROS are a potential source of damage to the vasculature (**Figure 1**) (Rifkind et al., 2003; Taniyama and Griendling, 2003). ROS by reacting with nitric oxide (NO) impair endothelial dependent vasorelaxation, which has a major effect on blood flow and oxygen delivery. Other established effects of ROS on the vasculature include an inflammatory response (Huertas et al., 2013), the increased expression of adhesion molecules like the vascular adhesion molecule (VCAM-1) and the intracellular adhesion molecule (ICAM-1), which result in monocyte adhesion contributing to atherosclerotic lesion formation (Dimmeler and Zeiher, 2000). In addition, ROS induce proliferation and migration of endothelial cells, mediate lymphocyte-activated tubulogenesis and mediate angiogenic growth factors like VEGF, all of which contribute to vascular remodeling (Staiculescu et al., 2014).

**FIGURE 2:**
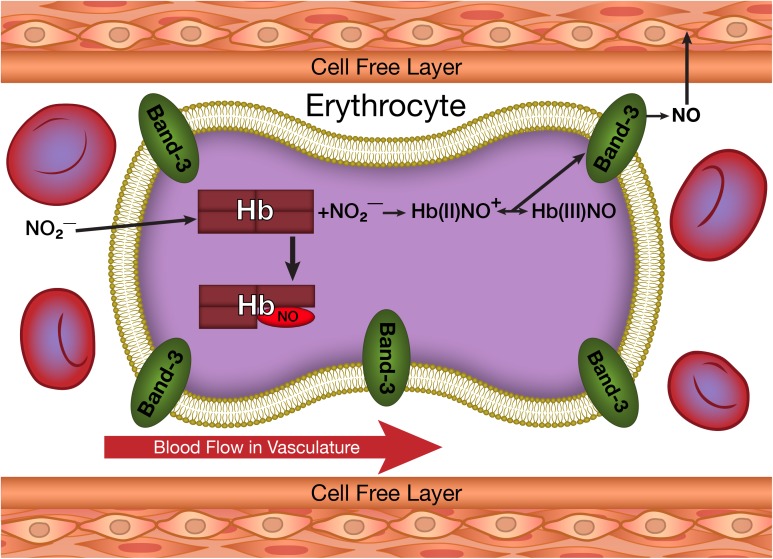
The production of NO from nitrite taken up by erythrocytes and its potential transfer to the vasculature.

An additional secondary reaction associated with hemoglobin involves the production of NO (**Figure 2**) by the reaction of deoxygenated heme groups with nitrite (Cosby et al., 2003; Nagababu et al., 2003b). Nitrite is produced in the circulation when NO released by the endothelium reacts with oxygen (Liu et al., 1998). This nitrite can be taken up by the erythrocyte and the vasculature. Nitrite in the vascular wall can be converted into NO under hypoxic conditions (Kleinbongard et al., 2006; Dalsgaard et al., 2007; Li et al., 2008; Aamand et al., 2009). In the erythrocyte, NO can also be generated by the hypoxic reaction of nitrite with deoxygenated hemoglobin (**Figure 2**). Another source of erythrocyte NO involves erythrocyte NO synthase (Kleinbongard et al., 2006). These pools of NO, if transmitted to the vasculature (**Figure 2**) can play a role in regulating dilatation and vascular tone (Ignarro et al., 1999).

Despite the potential vascular effects of both ROS and NO, cytoplasmic reactions within the erythrocyte are expected to completely neutralize any ROS (**Figure 1**) or NO (**Figure 2**) formed in the erythrocyte. Superoxide dismutase reacts with any superoxide formed, while catalase and glutathione peroxidase react with any hydrogen peroxide formed (Nagababu et al., 2003a). NO has a very high affinity for deoxygenated hemoglobin chains (Lancaster, 1994) and readily reacts with the oxygenated chains (Kelm et al., 1997). Therefore, the erythrocyte is not expected to be a source for vascular ROS or NO.

We have, however, demonstrated that significant amounts of ROS generated by hemoglobin autoxidation can be released from the erythrocyte, despite the high levels of erythrocyte antioxidants. This release was explained by a unique conformation for partially liganded hemoglobin formed under hypoxic conditions. This conformational change both increases the rate of autoxidation of oxygenated hemoglobin producing superoxide (Abugo and Rifkind, 1994) and enhances the binding of the partially liganded hemoglobin to the membrane band 3 (Cao et al., 2009) facilitating the release of the produced superoxide from the erythrocyte without being scavenged by erythrocyte antioxidant enzymes.

The direct formation of NO when nitrite reacts with deoxygenated hemoglobin would result in the immediate neutralization of the NO by reactions with hemoglobin (see above). We, however, found that the reaction of nitrite with deoxyhemoglobin produces two hemoglobin associated intermediates prior to the formation of NO (Salgado et al., 2009) The same conformational change for partially liganded hemoglobin that enhances the binding of partially oxygenated hemoglobin to the membrane facilitating the release of superoxide (ROS) also increases the affinity of the nitrite-reacted hemoglobin for band 3 on the erythrocyte membrane and enhances the formation of NO from one of these nitrite-reacted hemoglobin intermediates (Salgado et al., 2015). These reactions facilitate the release of NO from the erythrocyte.

Once released from the erythrocyte, possible effects of ROS and NO on the vasculature and other cells in the circulation need to be considered.

## The Reaction of Nitrite With Deoxyhemoglobin

The initial reaction in the erythrocyte involves the binding of nitrite to the hemoglobin deoxygenated hemes. This complex is then converted to an intermediate (Salgado et al., 2009) Hb(II)NO^+^. This intermediate can share an electron with the heme iron and the beta–93 thiol group producing a hybrid intermediate with properties of Hb(II)NO^+^, Hb(III)NO and ⋅SHb(II)NO (Salgado et al., 2011). The electron delocalization produces a relatively stable complex that retains the nitrite in a form that is not neutralized by irreversible reactions with hemoglobin. This complex can under proper conditions release NO. The Hb(III)NO can directly release NO and the Hb(II)NO^+^ can release NO by a nucleophilic reaction involving the distal histidine in the same way that oxyhemoglobin releases superoxide (Salgado et al., 2015). These reactions provide a pool of hemoglobin that can potentially release NO.

Since the reaction with nitrite occurs with hemoglobin that is appreciably deoxygenated, the nitrite-reacted-hemoglobin has the same properties as partially liganded hemoglobin. Therefore, it has an increased affinity for band 3 of the erythrocyte membrane. This results in a pathway for releasing NO from the erythrocyte when nitrite reacts with deoxygenated hemoglobin that is analogous to the pathway for the release of ROS from partially oxygenated hemoglobin.

## The Distinct Hemoglobin Conformational Change for Partially Liganded Hemoglobin That Facilitates the Release of ROS and NO From the Erythrocyte

### The Involvement of Ligand Induced Fluctuations

Hemoglobin consists of four subunits, two α-chains and two β-chains. Oxygen binds to the distal side of the heme groups with a histidine bound directly to the opposite proximal side of the heme. The four subunits are arranged tetragonally with two distinct interfaces between the α and β chains, the α_1_β_1_ and α_1_β_2_ interfaces.

The structural studies of hemoglobin have focused on the distinct properties of fully oxygenated (R-state) hemoglobin (Shaanan, 1983) and fully deoxygenated (T-state) hemoglobin (Fermi et al., 1984), with cooperativity explained by the cooperative structural changes detected by X-ray diffraction studies that take place when the T-state converts to the R-state (Perutz, 1970; Shaanan, 1983; Fermi et al., 1984). The structural changes detected by X-ray studies primarily involve the proximal side of the heme that undergoes an altered configuration when oxygen is bound. These changes are transmitted to the α_1_β_2_ interface, which undergoes a rearrangement that alters the quaternary conformation from that of the T-state to that of the R-state. It is this quaternary change in conformation that is considered responsible for the cooperative binding of oxygen, which results in the efficient transfer of oxygen to the tissues. Although other studies have suggested other conformational states for hemoglobin (Makino and Sugita, 1982; Smith et al., 1987; Perrella et al., 1990), these have also focused on the proximal side of the heme and the α_1_β_2_ interface.

We have, however, demonstrated alterations in the conformation of hemoglobin that involve perturbations of the distal pocket that are transmitted across the α_1_β_1_ interface to other subunits (Levy et al., 1992). These changes were originally demonstrated using valency hybrids where some subunits had Fe(II) hemes that bind oxygen and carbon monoxide, while other subunits had Fe(III) hemes that do not bind oxygen or carbon monoxide but can undergo configurational changes that are readily detected by electron paramagnetic resonance. Oxygen and carbon monoxide, which can both bind to the Fe(II) subunits, have different configurations when bound resulting in distinctly different interactions with the distal heme pocket. When the oxygen in the Fe(II) chains were replaced by carbon monoxide, these distal pocket changes were found to be transmitted to the Fe(III) subunit across the α_1_β_1_ interface. Unlike the perturbations of the α_1_β_2_ interface involved in the T to R quaternary conformational change, the α_1_β_1_ interface does not undergo any noticeable structural changes. Instead, we are dealing with fluctuations transmitted through the interface from one subunit to another. These primarily dynamic changes are not readily detected by X-ray diffraction studies.

### Dynamic Coupling Between Subunits in Partially Oxygenated Hemoglobin

Our initial studies involved R-state valency hybrids where perturbations were induced by replacing oxygen with carbon monoxide. The demonstration that changes induced in the distal pocket by different ligands are transmitted across the α_1_β_1_ interface implies closely coupled dynamic interactions between subunits. This same coupling would be expected to induce changes in T-state hemoglobin, with a more closely knit distal pocket, when oxygen is initially bound to deoxygenated hemoglobin producing partially oxygenated hemoglobin. This predicts that partially liganded hemoglobin, where one of the two subunits linked by the α_1_β_1_ interface has a ligand and the other chain is unliganded, should result in perturbations in the ligand pocket that should be transmitted between subunits.

This process was originally demonstrated in studies involving the changes in the rates of hemoglobin autoxidation as a function of the partial pressure of oxygen (Abugo and Rifkind, 1994; Balagopalakrishna et al., 1996). We, thus, found a dramatic increase in the rate of autoxidation for partially oxygenated hemoglobin (Abugo and Rifkind, 1994). This was interpreted in terms of a dramatic increase in fluctuations in the subunit with oxygen present as a result of the absence of a ligand in the coupled subunit. These increased fluctuations facilitate a nucleophilic interaction of the distal histidine with the bound oxygen resulting in the production of superoxide (**Figure 1**) (Balagopalakrishna et al., 1996).

More recently an analogous process was postulated to explain the release of NO from hemoglobin when deoxyhemoglobin (deoxyHb) reacts with nitrite (Nagababu et al., 2003b; Salgado et al., 2015). The initial reaction of nitrite with deoxyHb produces a hybrid intermediate with properties of Hb(II)NO^+^, Hb(III)NO and ⋅SHb(II)NO (Salgado et al., 2009, 2011). The unique properties found for partially oxygenated hemoglobin are expected to be similar to those of any partially liganded hemoglobin. The hybrid intermediate produced when low levels of nitrite react with deoxyHb, is a partially liganded hemoglobin with a heme reacted with nitrite instead of oxygen. The same nucleophilic displacement reaction, which transfers an electron from the Fe(II) heme to the bound oxygen producing superoxide, will transfer an electron from the Fe(II) heme to the bound NO^+^ producing NO (Salgado et al., 2015). This reaction is expected to result in the release of NO from hemoglobin (**Figure 2**) in the same way that autoxidation results in the release of ROS (**Figure 1**). The Fe(III)NO component of the hybrid intermediate has a very low affinity for the NO bound and will also release NO.

### Changes in the α_1_β_1_ Interface Increase Membrane Affinity of Hemoglobin

The superoxide and NO released from hemoglobin would be expected to be neutralized by antioxidant enzymes (superoxide dismutase) and irreversible reactions with hemoglobin, respectively. We have, however, found that ROS generated in the erythrocyte can be transferred to the vasculature (Kiefmann et al., 2008). Such a transfer would have to involve the ROS from the erythrocyte membrane avoiding neutralization by the cytosolic antioxidant enzymes. A role for hemoglobin binding to band 3 of the erythrocyte membrane was further confirmed by the finding that blocking the band 3 binding site inhibits the transfer of ROS to the vasculature (Huertas et al., 2013).

Our studies with nitrite-reacted hemoglobin have demonstrated that partially liganded hemoglobin (in this case the nitrite-reacted deoxygenated hemoglobin) has a higher affinity for the erythrocyte membrane (presumably band 3) than deoxyhemoglobin, known to have a higher affinity than oxyhemoglobin (Cao et al., 2009; Salgado et al., 2015). We have, thus, shown that when erythrocytes are deoxygenated, the low levels of nitrite-reacted hemoglobin present are in large part associated with the limited membrane binding sites. This occurs even though there is a very large excess of deoxygenated hemoglobin not reacted with nitrite (Salgado et al., 2015). To rule out a specific effect involving nitrite reacted hemoglobin, we found that the affinity of nitrite reacted hemoglobin for the membrane was appreciably reduced when the sample was oxygenated. These results indicate that the increased affinity is associated with a partially liganded hemoglobin state irrespective of the ligand bound to the hemoglobin

**FIGURE 3:**
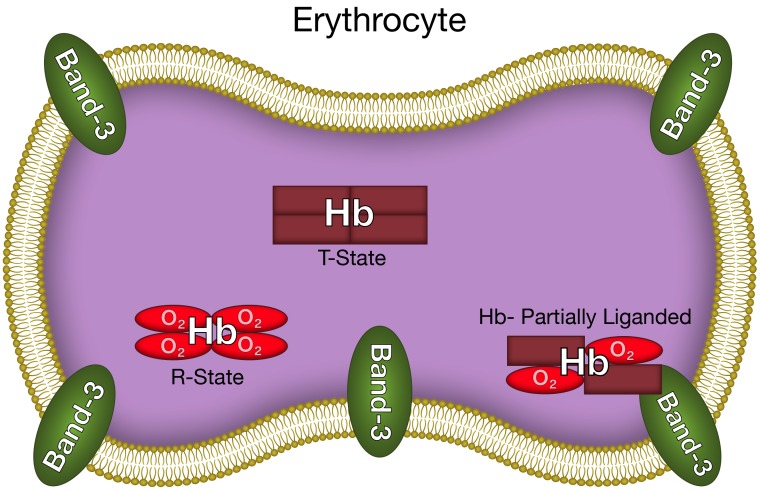
The altered hemoglobin conformation of partially liganded hemoglobin that results in enhanced binding to the erythrocyte membrane.

This increased membrane-band 3 affinity for partially liganded hemoglobin can be attributed to the same increased fluctuations across the α_1_β_1_ interface (Levy et al., 1992) that have been shown to increase the release of ROS and NO. The hemoglobin interfaces are involved in the binding of hemoglobin to the erythrocyte membrane band 3 (Chetrite and Cassoly, 1985). Interface interactions have been attributed to the significant increase in membrane affinity for T-state deoxyHb relative to R-state oxyhemoglobin (oxyHb). The increased fluctuations being transmitted across the α_1_β_1_ interface for partially liganded hemoglobin can also affect the hemoglobin membrane-band 3 interactions. Such an interaction would explain the enhanced affinity of partially liganded hemoglobin for band 3 of the erythrocyte membrane (Salgado et al., 2015). We can, therefore, conclude that the same conformational change responsible for the enhanced formation of superoxide and NO from partially liganded hemoglobin, also results in a dramatic increase in the affinity of hemoglobin for the erythrocyte membrane (**Figure 3**).

### The Release of Superoxide and NO From Erythrocytes

As indicated above the erythrocyte cytoplasmic antioxidants and hemoglobin react with any ROS or NO released from hemoglobin. However, the dramatic increase in binding of partially oxygenated hemoglobin and nitrite-reacted hemoglobin to the membrane (**Figure 3**) results in a significant fraction of any released superoxide or NO diffusing out of the erythrocyte without being neutralized (**Figures 1**, **2**). The same conformational change enhances the formation of superoxide and NO (see above). For the NO reaction, by a determination of the NO in the gas phase above a solution of nitrite reacted hemoglobin, we have actually shown, that the altered interface fluctuations involved in binding hemoglobin to the membrane further enhances the nucleophilic displacement that enhances the release of NO from the erythrocyte (Salgado et al., 2015).

The unique conformation of partially liganded hemoglobin, involving fluctuations transmitted across the α_1_β_1_ interface between adjacent subunits increase band 3 membrane interactions of hemoglobin (**Figure 3**) and nucleophilic interactions of the distal histidine with ligands bound to the ferrous heme (**Figures 1**, **2**). These changes provide a mechanism for the release of NO and ROS from the erythrocyte.

## Transfer of Reactive ROS and NO From the Erythrocyte to the Vasculature

It was demonstrated that under hypoxic conditions, the conformation of partially liganded hemoglobin facilitates the release of ROS and/or NO from the erythrocyte (see above). For ROS, we have demonstrated that the released ROS is transferred into capillary venules (Huertas et al., 2013; Kiefmann et al., 2008). Whether the transfer is due to diffusion across the narrow cell free layer present in capillaries (Ng et al., 2015) or a transient adherence of hypoxic erythrocytes to the vasculature is being investigated. It has further been demonstrated that this transfer induces an inflammatory response (Huertas et al., 2013) that can modulate vascular function.

Nitric oxide released from the erythrocyte can interact with platelets in the circulation inhibiting platelet activation (Wang et al., 1998). The resultant inhibition of platelet adhesion to the vascular endothelium can affect vascular function. It has also been reported that NO generated in the erythrocyte can dilate small mesenteric arteries (Ulker et al., 2013) suggesting that under certain conditions the highly reactive NO released from erythrocytes can interact directly with the vasculature.

These findings are supported by a computational study utilizing a multicellular model to simulate the transport of NO generated by nitrite from erythrocytes to the vasculature and the smooth muscle cells in arterioles (Chen et al., 2008). In this model, free diffusion of NO released in the cells provided only 0.04 pM NO in the smooth muscle cells, which is not expected to cause any vasodilatation. However, a membrane associated mechanism that protects NO bioactivity and facilitates its export out of the erythrocyte increases the level of NO that can react with the smooth muscle cells by 3 orders of magnitude to ∼ 43 pM and can cause vasodilatation. This dramatic increase took into account the diffusion of released NO back into the erythrocyte, where it can be scavenged by hemoglobin. Our studies involving the release of NO from hemoglobin bound to band 3 of the erythrocyte membrane reflects such a membrane-associated mechanism and is consistent with potential effects on the vasculature. The study by Liu et al. (2007) that concluded that NO generated in the erythrocyte cannot exert any physiological effects were limited to free diffusion of NO released in the cell with very high membrane permeability. Under such conditions interactions with the erythrocyte membrane were eliminated. Experiments are being planned to confirm the transfer of NO from the erythrocyte to the vasculature in the same way that we were able to show that ROS are transferred from the erythrocyte to the vasculature.

## Conclusion

A completely new perspective in our understanding of interactions between erythrocytes and the vasculature is provided by these studies. A distinct conformation of hemoglobin under hypoxic conditions provides a mechanism for the release of NO and ROS from the erythrocytes (**Figure 3**). Furthermore, at least in arterioles and narrow capillaries, these molecules can potentially be transferred to the vasculature (**Figures 1**, **2**).

For ROS, this transfer into the vasculature under hypoxic conditions has been demonstrated (Kiefmann et al., 2008). Additional studies are required to demonstrate that NO released into the lumen from the erythrocyte can diffuse into the smooth muscle cells of arterioles to induce vasodilatation.

We are also investigating a possible role for erythrocyte adherence to the vasculature. Such adherence that is involved in many pathological conditions (Setty and Stuart, 1996) provides a mechanism for increasing the time the erythrocyte is in contact with the endothelium. Such an interaction would also be expected to increase the transfer of both NO and ROS to the vasculature.

For the release of ROS and transfer of ROS to the vasculature, hemoglobin must be partially oxygenated (**Figure 1**). However, the hemoglobin conformation that triggers membrane binding and the potential transfer of NO utilizes a partially liganded hemoglobin involving the nitrite reacted intermediate and does not require an oxygen bound to the hemoglobin (**Figure 2**). This process is therefore able to deliver NO to the vasculature even under very low partial pressures of oxygen where NO synthase is unable to generate NO. The transfer of NO from the erythrocyte to the vasculature can, therefore, play an important role in regulating vascular function when an impaired vasculature and/or reduced levels of oxygen limit the production of NO.

## Author Contributions

JR was responsible for all the research that led to this hypothesis originally as section chief of Molecular Dynamics at the National Institute on Aging. While at Johns Hopkins these ideas were finalized and the manuscript was written. JM was responsible for the figures and the bibliography. He also helped formulate the hypothesis and write the manuscript. EN played a major role in our understanding of the formation of nitric oxide and reactive oxygen species by erythrocytes. MS performed crucial experiments on the formation and release of nitric oxide formed in erythrocytes due to the reaction of nitrite with deoxyhemoglobin. ZC did crucial experiments involving the association of partially oxygenated hemoglobin and nitrite reacted hemoglobin with the erythrocyte membrane. This binding is what facilitates the release of nitric oxide and reactive oxygen species from the erythrocyte.

## Conflict of Interest Statement

The authors declare that the research was conducted in the absence of any commercial or financial relationships that could be construed as a potential conflict of interest.
